# All-*trans* retinoic acid as adjunct to intensive treatment in younger adult patients with acute myeloid leukemia: results of the randomized AMLSG 07-04 study

**DOI:** 10.1007/s00277-016-2810-z

**Published:** 2016-10-03

**Authors:** Richard F. Schlenk, Michael Lübbert, Axel Benner, Alexander Lamparter, Jürgen Krauter, Wolfgang Herr, Hans Martin, Helmut R. Salih, Andrea Kündgen, Heinz-A. Horst, Peter Brossart, Katharina Götze, David Nachbaur, Mohammed Wattad, Claus-Henning Köhne, Walter Fiedler, Martin Bentz, Gerald Wulf, Gerhard Held, Bernd Hertenstein, Hans Salwender, Verena I Gaidzik, Brigitte Schlegelberger, Daniela Weber, Konstanze Döhner, Arnold Ganser, Hartmut Döhner, for the German-Austrian Acute Myeloid Leukemia Study Group

**Affiliations:** 1 Department of Internal Medicine III, University Hospital of Ulm, Albert-Einstein-Allee 23, 89081 Ulm, Germany; 2 National Center for Tumor Diseases (NCT), German Cancer Research Center, Heidelberg, Germany; 3 Department of Hematology and Oncology, University Hospital of Freiburg, Freiburg, Germany; 4 Division of Biostatistics, German Cancer Research Center, Heidelberg, Germany; 5 Department of Oncology and Hematology, Klinikum Braunschweig, Braunschweig, Germany; 6 Department of Hematology, Hemostasis, Oncology and Stem Cell Transplantation, Hannover Medical School, Hannover, Germany; 7 Department of Medicine III, Johannes Gutenberg-University Mainz, Mainz, Germany; 8 Department of Internal Medicine III, University of Regensburg, Regensburg, Germany; 9 Department of Internal Medicine II, University Hospital, Frankfurt, Germany; 10 Department of Hematology and Oncology, Eberhard-Karls University, Tübingen, Germany; 11 Department of Hematology, Oncology and Clinical Immunology, Heinrich-Heine-University Düsseldorf, Düsseldorf, Germany; 12 Department of Internal Medicine II, University Hospital Schleswig-Holstein Campus Kiel, Kiel, Germany; 13 Department of Internal Medicine III, University Hospital of Bonn, Bonn, Germany; 14 Department of Internal Medicine III, Technical University of Munich, Munich, Germany; 15 Department of Internal Medicine V, University Hospital Innsbruck, Innsbruck, Austria; 16 Department of Hematology, Oncology and Stem Cell Transplantation, Klinikum Essen Süd, Essen, Germany; 17 Department of Oncology and Hematology, Klinikum Oldenburg, Oldenburg, Germany; 18 Department of Internal Medicine II, University Medical Center Hamburg-Eppendorf, Hamburg, Germany; 19 Department of Internal Medicine III, Städtisches Klinikum Karlsruhe, Karlsruhe, Germany; 20 Department of Hematology and Oncology, University Hospital of Göttingen, Göttingen, Germany; 21 Department of Internal Medicine I, University Hospital of Saarland, Homburg, Germany; 22 Department of Internal Medicine I, Klinikum Bremen Mitte, Bremen, Germany; 23 Department of Hematology/Oncology, Asklepios Klinik Altona, Hamburg, Germany; 24 Institute of Human Genetics, Hannover Medical School, Hannover, Germany

**Keywords:** Acute myeloid leukemia, All-*trans* retinoic acid, Nucleophosmin-1

## Abstract

The aim of this clinical trial was to evaluate the impact of all-*trans* retinoic acid (ATRA) in combination with chemotherapy and to assess the *NPM1* status as biomarker for ATRA therapy in younger adult patients (18–60 years) with acute myeloid leukemia (AML). Patients were randomized for intensive chemotherapy with or without open-label ATRA (45 mg/m^2^, days 6–8; 15 mg/m^2^, days 9–21). Two cycles of induction therapy were followed by risk-adapted consolidation with high-dose cytarabine or allogeneic hematopoietic cell transplantation. Due to the open label character of the study, analysis was performed on an intention-to-treat (ITT) and a per-protocol (PP) basis. One thousand one hundred patients were randomized (556, STANDARD; 544, ATRA) with 38 patients treated vice versa. Median follow-up for survival was 5.2 years. ITT analyses revealed no difference between ATRA and STANDARD for the total cohort and for the subset of *NPM1*-mutated AML with respect to event-free (EFS; *p* = 0.93, *p* = 0.17) and overall survival (OS; *p* = 0.24 and *p* = 0.32, respectively). Pre-specified PP analyses revealed better EFS in *NPM1*-mutated AML (*p* = 0.05) and better OS in the total cohort (*p* = 0.03). Explorative subgroup analyses on an ITT basis revealed better OS (*p* = 0.05) in ATRA for genetic low-risk patients according to ELN recommendations. The clinical trial is registered at clinicaltrialsregister.eu (EudraCT Number: 2004-004321-95).

**Electronic supplementary material:**

The online version of this article (doi:10.1007/s00277-016-2810-z) contains supplementary material, which is available to authorized users.

## Introduction

All-*trans* retinoic acid (ATRA) in combination with chemotherapy or arsenic trioxide (ATO) has revolutionized the treatment of acute promyelocytic leukemia (APL) [1, 2]. However, early preclinical studies also provided a rationale for the use of ATRA in non-APL acute myeloid leukemia (AML) [3–9]. *In vitro* studies showed efficacy of ATRA in non-APL AML cell lines and primary AML blasts, especially in co-treatment of leukemic blasts with cytarabine [3–5] or idarubicin [6].

These *in vitro* experiments provided important evidence that the addition of ATRA to cytarabine or idarubicin only increases the killing of clonogenic cells when ATRA is administered after exposure to the cytotoxic drug [3–7]. Besides a shortening of the BCL2 half-life, which has been implicated as a resistance mechanism in AML [3, 5, 8, 10], an additional potential pathophysiological mechanism of the anti-leukemic activity of ATRA was described by Balusu et al. in AML with mutant *NPM1* [11]. NPM1 levels attenuated by ATRA selectively induced apoptosis and sensitized AML with mutant *NPM1* to treatment with ATRA and cytarabine [11]. More recently, two groups showed that the combination of ATRA and ATO synergistically induced proteasomal degradation of mutant NPM1, leading to growth arrest, differentiation and apoptosis [12, 13].

Based on the promising *in vitro* data, several clinical trials evaluated ATRA in combination with chemotherapy in non-APL AML. Encouraging data from a phase II trial combining low-dose cytarabine with ATRA in 33 patients ineligible for intensive therapy [14] triggered larger up-front randomized trials [15–19]. The results from these randomized studies have been contradictory, with the majority reporting negative results. In the study by Estey et al. of 215 patients with high-risk myelodysplastic syndrome or AML older than 71 years, there was no effect of ATRA in multivariable analysis, but a significantly better overall survival was found in univariable analyses for patients treated in the ATRA arms [15]. The British Medical Research Council (MRC) performed three randomized trials, one in younger patients receiving intensive first-line treatment (MRC AML12, *n* = 1097) [16], one in medically unfit patients (MRC AML14, *n* = 207) [17], and one in high-risk refractory or relapsed patients (MRC AML-HR, *n* = 362) [18], without showing a significant effect of ATRA on any endpoint analyzed.

In these trials showing negative results, ATRA consistently was started simultaneously [16–18] or before initiation of chemotherapy [15]. In contrast, in our AMLHD98B trial of 242 older patients, ATRA was started at the end of chemotherapy in accordance with the *in vitro* data [3–7, 19]. In this trial, patients randomized to the ATRA arm had a significantly higher complete remission (CR) rate, better event-free and overall survival [19]. In a subsequent subgroup analysis of the up-front randomized patients (206 of 242), the genotype mutated *NPM1* in the absence of *FLT3* internal tandem duplication (ITD) emerged as a predictive marker for the beneficial effect of ATRA [20]. However, similar biomarker analyses on selected patients (592 of 1075) of the MRC AML 12 trial again did not reveal a beneficial clinical effect of ATRA in any of the analyzed subgroups [21]. Although not statistically significant but consistent with the results of the AMLHD98B trial, a better relapse-free and overall survival was present in patients exhibiting the genotype mutated *NPM1* in the absence of *FLT3*-ITD who had been randomized to the ATRA arm (estimated hazard ratio for overall survival, 0.70; 95 % confidence interval [CI], 0.42–1.16) [21].

In 2004, we initiated the up-front randomized AMLSG 07-04 four-arm study evaluating in a two-by-two factorial design ATRA and valproic acid (VPA) as adjunct to intensive induction and consolidation therapy. In 2006, the protocol was amended and the randomization for VPA was terminated based on excessive hematologic toxicity of VPA in combination with chemotherapy, which was similarly noted in older patients [22]. Here, we report the results of the upfront randomization for ATRA in 1100 younger adult patients.

## Patients and methods

### Patients

Patients aged between 18 and 60 years with newly diagnosed AML including *de novo* AML, secondary AML with a preceding history of myelodysplastic or myeloproliferative disorder (sAML) and therapy-related AML following treatment of a primary malignancy (tAML), as defined by the WHO 2001 classification were eligible for the trial [23]. Patients with acute promyelocytic leukemia (APL) as well as patients with concomitant renal (creatinine > 1.5 x upper normal serum level), liver (bilirubin, AST or AP > 2 x upper normal serum level) or cardiac dysfunction (New York Heart Association III/IV), uncontrolled infectious disease, primary coagulation disturbance or performance status (ECOG) >2 were excluded. Written informed consent was obtained from all patients. The protocol was approved by the lead Ethics Review Committee and registered at clinicaltrialsregister.eu (EudraCT Number: 2004-004321-95) and clinicaltrials.gov (NCT00151242).

### Cyto- and molecular genetics

Chromosome banding analysis was performed centrally in the two AMLSG Laboratories for Cytogenetics (Hannover, Ulm). Karyotypes were designated according to the International System for Human Cytogenetic Nomenclature [24]. Leukemia samples were analyzed for mutations in *FLT3* (ITDs and tyrosine kinase domain [TKD] mutations at codons D835/I836), *NPM1*, *CEBPA*, *DNMT3A, RUNX1*, *IDH1/2*, *ASXL1* and *CEBPA* as previously described [20, 25–29].

### Study design

#### Induction therapy

From August 2004 to January 2006, patients were randomized in a two-by-two factorial design to receive induction chemotherapy with or without ATRA and with or without VPA resulting in four arms, ATRA, ATRA-VPA, VPA and STANDARD. In January 2006, randomization for VPA was terminated due to increased hematologic toxicity whereas randomization for ATRA was carried forward. Induction therapy consisted of 2 cycles ICE (idarubicin, 12 mg/m^2^ i.v., days 1, 3 and 5; cytarabine, 100 mg/m^2^ cont. i.v., days 1–7; etoposide 100 mg/m^2^ i.v., days 1–3) or the same chemotherapy plus ATRA (ATRA p.o., 45 mg/m^2^, days 6–8 and 15 mg/m^2^, days 9–21). Patients achieving a CR or partial remission (PR) after the first induction received a second cycle according to their initial randomization with a reduced dosage of idarubicin (12 mg/m^2^, days 1 and 3).

#### Consolidation therapy

Patients with high-risk AML defined either by high-risk cytogenetics or induction failure [30] were assigned to receive an allogeneic hematopoietic cell transplantation (HCT) from a matched related (MRD) or unrelated donor (MUD). Starting from December 2006, AML exhibiting a *FLT3*-ITD was also categorized as high risk [25]. All other patients were assigned either to three cycles of high-dose cytarabine (HiDAC) from August 2004 to November 2006 with cytarabine 3 g/m^2^ bid, days 1, 3 and 5, and from November 2006 with a condensed schedule with application of cytarabine 3 g/m^2^ bid, days 1, 2 and 3. If an MRD was available, an allogeneic HCT was intended in first CR in all patients except those with core-binding factor AML.

### Definition of response criteria, survival endpoints and hematologic recovery

In accordance with standard criteria, CR was defined as less than 5 % bone marrow blasts, an absolute neutrophil count of 1.0 G/L or higher, a platelet count of 100 G/L or higher, no blasts in the peripheral blood and no extramedullary leukemia; CR with incomplete blood count recovery (CRi) was characterized as CR except for residual neutropenia (neutrophils <1.0 G/L) or thrombocytopenia (platelets <100 G/L) [31]. Relapse was defined as more than 5 % bone marrow blasts unrelated to recovery from the preceding course of chemotherapy or new extramedullary leukemia in patients with previously documented CR.

Event-free survival (EFS), relapse-free survival (RFS) and overall survival (OS) were defined as recommended [31]. Times to leukocyte, neutrophil and platelet recovery were measured from the first day of chemotherapy of each cycle until the first day with values more than or equal to 1, 0.5 and 20 G/L for white blood cells (WBC), neutrophils and platelets, respectively. Toxicities were defined and graded according to the National Cancer Institute (NCI) Common Toxicity Criteria, version 2.0.

### Statistical analysis

Pairwise comparisons between patient subgroups were performed by the Mann-Whitney or Kruskal-Wallis test for continuous variables and by Fisher’s exact test for categorical variables. Univariable and multivariable logistic regression models were applied to investigate the influence of covariates on response to induction therapy.

The analysis were performed on an intention-to-treat (ITT) according to initial randomization result and a per protocol (PP) basis according to received treatment. The primary endpoint of the study was EFS; secondary endpoints were OS, RFS, therapy-related toxicity and their correlation with the study drug. The median duration of follow-up was calculated by the reverse Kaplan-Meier estimate [32]; the Kaplan-Meier method was used to estimate the distributions of EFS, RFS and OS. Survival distributions were compared using the log-rank test. Multivariable Andersen-Gill regression models were used to evaluate prognostic variables including allogeneic HCT as a time-dependent covariable [33]. In addition, the following variables were evaluated in multivariable regression models: WBC (median-dichotomized), age, gender, genetic-risk group according to European LeukemiaNet (ELN) recommendations (favorable, intermediate-1, intermediate-2, adverse) [34], type of AML (*de novo*, sAML/tAML), randomization (STANDARD, ATRA), *FLT3-*TKD, *FLT3-*ITD, *NPM1*, biallelic mutated *CEBPA*, *DNMT3A*; *IDH1*, *IDH2*, *RUNX1* and *ASXL1* mutational status and VPA (received, not received). Pre-specified subset analyses, according to the *NPM1* and the combined *NPM1* and *FLT3*-ITD mutational status, were performed for all endpoints. Missing data were replaced by 50 imputations using multivariate imputations by chained equations applying predictive mean matching [35]. Backward selection applying a stopping rule based on a *p* value of 0.50 was used in multivariable regression models to exclude redundant or unnecessary variables [35].

All statistical analyses were performed with the statistical software environment R, version 3.0.1, using the R packages rms, version 3.6-3, and cmprsk, version 2.2-2 [36].

## Results

### Patients and baseline characteristics

A total of 1229 patients were registered, 809 were randomized first within the framework of the cooperative German AML Intergroup Study [37] in a ratio 1:10 into a common standard arm (*n* = 85) or the study group specific protocol (*n* = 724), and thereafter, 420 patients were directly registered for the AMLSG 07-04 protocol. Of 1144 randomized patients, 44 were excluded due to violation of in-/exclusion criteria (*n* = 29), no informed consent (*n* = 10) or other reasons (*n* = 5).

Between August 2004 and January 2006, patients were assigned to one of four arms according to the two-by-two factorial design, ATRA (*n* = 97), ATRA-VPA (*n* = 91), VPA (*n* = 95) and STANDARD (*n* = 98). After termination of the VPA-randomization, additional *n* = 719 patients were randomized for ATRA resulting in 544 patients in the ATRA (ATRA) and 556 in the STANDARD arm of the study (Table 1). Table 1 shows patient demographics and presenting laboratory and genetic characteristics by up-front randomization for ATRA. Patients in ATRA were characterized by significantly lower WBC (*p* = 0.003) and peripheral blast percentage (*p* = 0.003) compared to patients in STANDARD. Nine randomized patients did not receive the scheduled therapy, due to death before start of induction therapy (ATRA, *n* = 4; ATRA-VPA, *n* = 2; VPA, *n* = 0; STANDARD, *n* = 3).

**Table 1:** Description of patient characteristics, clinical and laboratory

	Standard	ATRA	*p* value
	*n* = 556	*n* = 544	
	No. (%)	No. (%)	
Age [years], median (range)	48.8 (18–61)	48.5 (18–61)	0.56
Gender [male], No. (%)	283 (50.9)	288 (52.9)	0.51
WBC [109/l], median (range)	16.0 (0.3–532)	9.2 (0.3–349)	0.003
Missing	3	8	
Platelets [10^9^/l], median (range)	52 (3–590)	58 (4–933)	0.11
Missing	4	8	
Hemoglobin [g/dL], (median, range)	9.1 (3.8–15.3)	9.2 (3.5–16.0)	0.77
Missings	3	7	
LDH [U/l], median (range)	445 (94–15098)	407.5 (84–6907)	0.18
Missings	8	8	
BM-blasts [%], median (range)*	75 (0–100)	70 (2–100)	0.14
Missings	30	33	
PB-blasts [%], median (range)	36 (0–100)	27 (0–100)	0.003
Missings	39	36	
Type of AML, No. (%)			0.99
De novo	484 (87)	473 (87)	
sAML	31 (5.6)	30 (5.5)	
tAML	40 (7.2)	40(7.4)	
Cytogenetic risk, No. (%)			0.58
CBF-AML	65 (12.8)	56 (11.0)	
Intermediate	336 (66.1)	338 (66.1)	
Adverse^30^	107 (21.1)	117 (22.9)	
Normal karyotype, No. (%)	246 (48.4)	248 (48.5)	0.99
Missings	48	33	
Biallelic mutated *CEBPA*, No. (%)	26 (5.3)	23 (4.8)	0.77
Missings	61	64	
*FLT3*-ITD, No. (%)	107 (20.2)	102 (20.1)	0.99
Missings	26	36	
*FLT3*-TKD, No. (%)	28 (5.3)	25 (5.0)	0.89
Missings	29	40	
Mutated *NPM1*, No. (%)	149 (29.2)	138 (27.8)	0.68
Missings	46	47	
Mutated *DNMT3A*, No. (%)	109 (21.5)	119 (23.9)	0.37
Missings	48	47	
Mutated *IDH1*, No. (%)	26 (6.1)	29 (6.8)	0.68
Missings	127	120	
Mutated *IDH2*R140, No. (%)	30 (7.0)	29 (6.9)	0.99
Mutated *IDH2*R172, No. (%)	12 (2.8)	11 (2.6)	
Missings	130	122	
Mutated *RUNX1*, No. (%)	39 (9.4)	32 (7.9)	0.54
Missings	139	140	
Mutated *ASXL1*, No. (%)	22 (5.3)	21 (5.1)	0.99
Missings	141	131	
ELN genetic risk group			0.80
Favorable risk, No. (%)	152 (30.3)	139 (28.0)	
Intermediate-2 risk, No. (%)	153 (30.5)	151 (30.4)	
Intermediate-2 risk, No. (%)	90 (17.9)	90 (18.1)	
Adverse risk, No. (%)	107 (21.3)	117 (23.5)	
Missings	54	47	

*Abbreviations*: *WBC* white blood count, *LDH* lactate-dehydrogenase, *BM* bone marrow, *PB* peripheral blood, *sAML* secondary AML after a preceding MDS; *tAML* treatment-related AML, *CBF- AML* core-binding factor AML, *CEBPA* CCAAT/enhancer binding protein alpha, *FLT3-ITD* FMS-like tyrosine kinase 3 gene internal tandem duplication, *FLT3-TKD* FMS-like tyrosine kinase 3 gene tyrosine kinase domain mutation, *NPM1* nucleophosmin, *DNMT3A* DNA (cytosine-5-)-methyltransferase 3 alpha, *IDH* Isocitrate dehydrogenase, *RUNX1* Runt-related transcription factor 1, *ASXL1* additional sex combs like 1, transcriptional regulator

*In case of BM blasts <20 %, diagnosis of AML was established based on extramedullary disease or PB blast >20 %

In spite of the initial randomization to ATRA, 19 patients did not receive ATRA due to the local physicians’ judgment. On the other hand, 19 patients received ATRA although randomized to STANDARD. According to the protocol and the open-label character of the study, ITT analyses followed by PP analyses were performed.

**Fig. 1:**
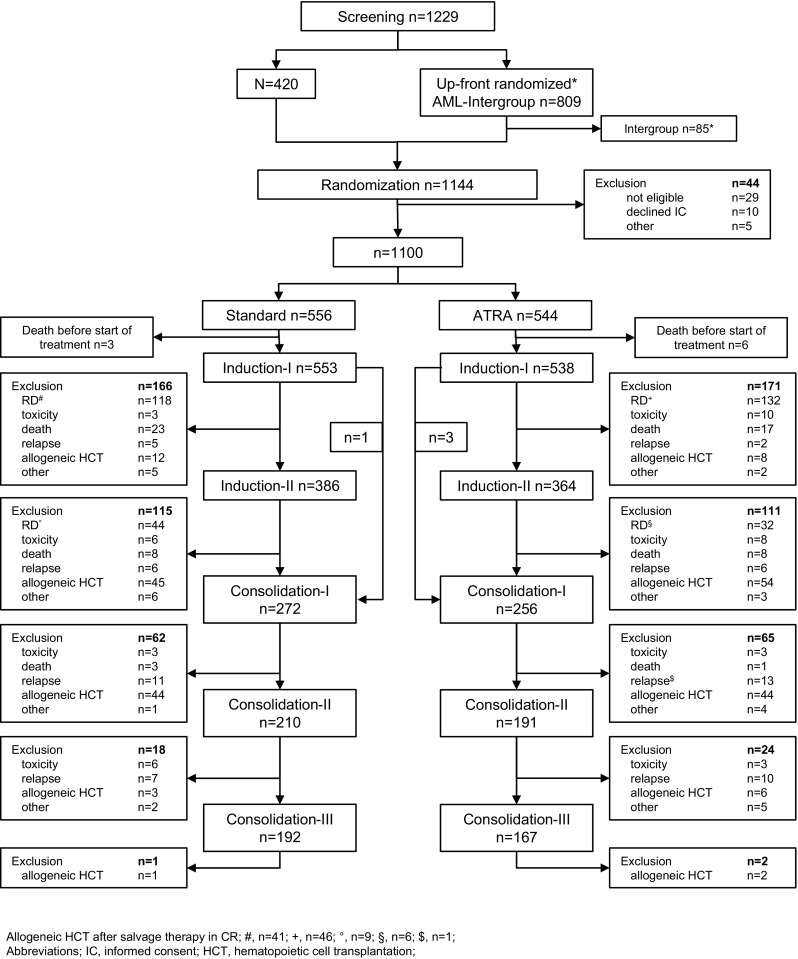
Flow chart on study conduct. Flow chart showing enrollment, program completion and/or drop-out according to the randomization result. Abbreviations: *IC* informed consent, *RD* refractory disease, *HCT* hematopoietic cell transplantation

The trial flow is summarized in the diagram according to CONSORT statement in Fig. 1.

### Response to induction therapy

After the first induction cycle, there was no significant difference between the two treatment arms on an ITT basis (ATRA, 50.9 %; STANDARD, 48.7 %) in achieving of CR/CRi (*p* = 0.47). In contrast, the PP analysis revealed a significantly (*p* = 0.03) higher CR/CRi rate in the ATRA (53.1 %) compared to the STANDARD arm (46.6 %). After double induction therapy, ITT analyses did not reveal a significant difference in CR/CRi rate (*p* = 0.95) between ATRA (73.3 %) and STANDARD (73.6 %), whereas in the PP analyses the CR/CRi rate in patients receiving ATRA (75.9 %) was in trend superior (*p* = 0.08) compared to patients in STANDARD (71.0 %). In the predefined *NPM1*-subsets (accounted for *FLT3*-ITD), no significant difference were identified.

Multivariable logistic regression analysis in all patients revealed no impact of ATRA on an ITT and PP basis (Supplementary Table 1).

In patients receiving ATRA, a low (2 %) but significantly (*p* = 0.04) increased rate of allergic reactions grade III/IV was reported compared to STANDARD (1 %). Of note, in STANDARD cardiac grade III/IV events were significantly (*p* = 0.03) more frequent (4 %) compared to ATRA (1.5 %). All other reported toxicities were equally distributed (Supplementary Table 2). No difference (*p* = 0.80) in death rate during double induction therapy was present between ATRA (5.7 %) and STANDARD (6.1 %). Recovery times of neutrophils (*p* = 0.61) and platelets (*p* = 0.70) after the first induction cycle were comparable between STANDARD and ATRA.

### Consolidation therapy

Allogeneic HCT in first CR after first or second induction therapy was performed in 57 and 62 patients in STANDARD and ATRA, respectively; in addition, 50 and 52 patients in STANDARD and ATRA with RD after induction therapy received allogeneic HCT in first CR following successful salvage therapy outside the protocol (Fig. 1).

One consolidation therapy with high-dose cytarabine was administered in 272 and 256 patients in STANDARD and ATRA, respectively; all three cycles of high-dose cytarabine were administered in 192 and 167 patients in STANDARD and ATRA, respectively. During consolidation therapy, 48 and 53 patients proceeded to allogeneic HCT in first CR in STANDARD and ATRA, respectively. In total, 155 and 167 patients received allogeneic HCT in first CR in STANDARD and ATRA, respectively. In addition, 149 patients received allogeneic HCT with active disease (STANDARD, *n* = 73; ATRA, *n* = 76) during first line therapy.

### Survival analyses

Estimated median follow-up for survival was 5.23 years (95 % CI, 5.02–5.37) without difference according the treatment arms (*p* = 0.69). Of the 1100 randomized patients, 808 achieved a first CR; of these, 397 relapsed, and overall, 562 died. After relapse, 88 and 90 % of the patients in STANDARD and ATRA were treated intensively (*p* = 0.43). Allogeneic HCT after relapse was performed in 231 patients (ATRA, *n* = 107; STANDARD, *n* = 124).

**Fig. 2:**
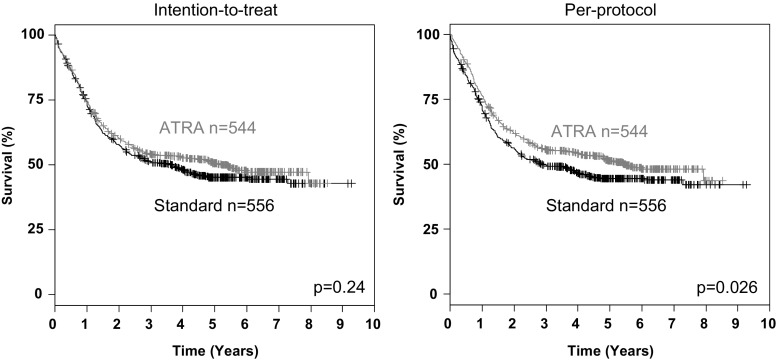
Survival analyses according to randomization according to intention-to-treat and per-protocol analysis

Univariable survival analyses on an ITT basis revealed no significant differences for EFS (*p* = 0.93), RFS (*p* = 0.25) and OS (*p* = 0.24, Fig. 2) according to the treatment arm. However, PP analyses showed a trend for superior EFS (*p* = 0.09) and a statistically significant better OS (*p* = 0.03, Fig. 2) for patients in ATRA compared to STANDARD, but no difference in RFS (*p* = 0.14). In the pre-defined predictive marker study, ITT analyses (Supplementary Figure 1) revealed no significant impact of ATRA in the *NPM1*-mutated and *NPM1*-wildtype subgroups for EFS (*p* = 0.17, *p* = 0.48) for RFS (*p* = 0.38, *p* = 0.28) and OS (*p* = 0.44 and *p* = 0.70, respectively), whereas PP analyses revealed significantly improved EFS for ATRA in the *NPM1*-mutated subgroup (*p* = 0.05, Supplementary Figure 2). Explorative analyses in molecularly defined subsets on OS revealed a significant beneficial effect on an ITT (Table 2) and PP basis (Supplementary Figure 3) of ATRA in patients in the ELN favorable-risk category (*p* = 0.05 and *p* = 0.05, respectively), and in particular, those patients exhibiting biallelic *CEBPA* mutations (*p* = 0.04 and *p* = 0.03, respectively).

**Table 2:**
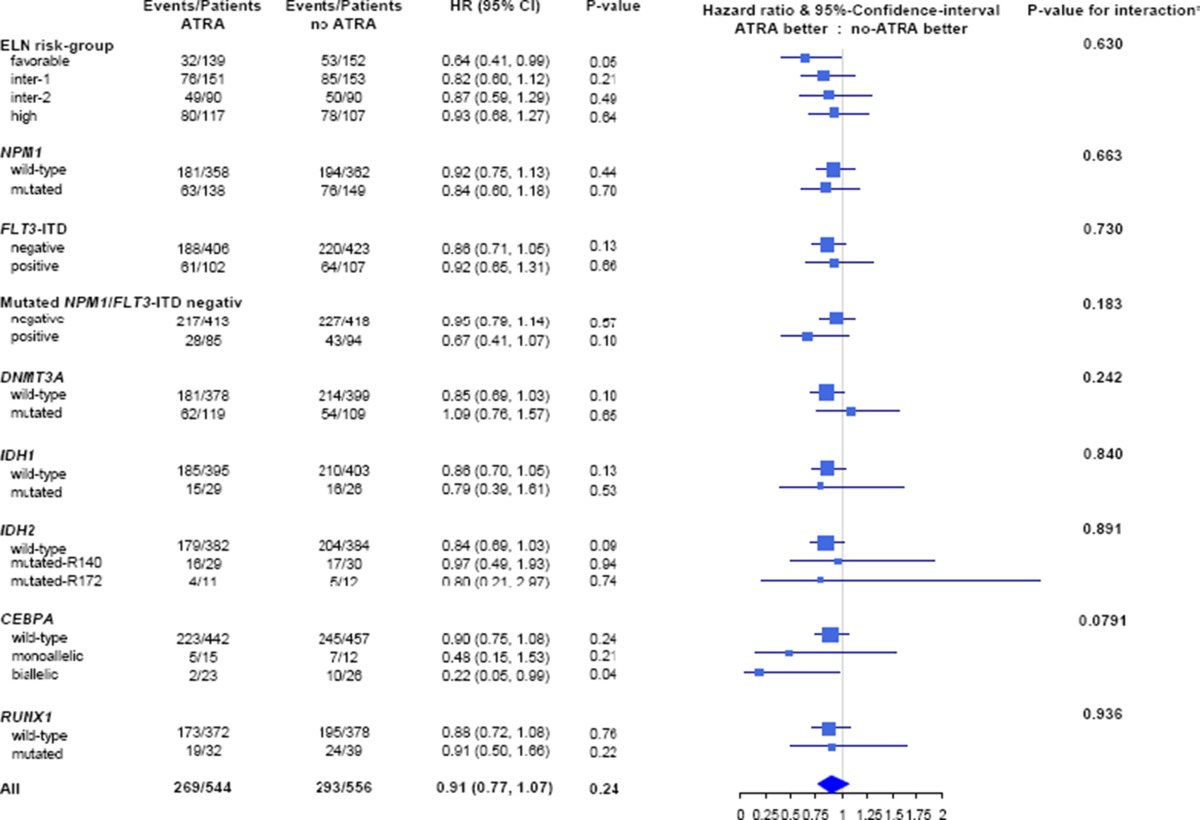
Stratified analyses of ATRA on an intention-to-treat basis by genetic risk group according to ELN recommendations and mutational status of NPM1, FLT3-ITD, DNMT3A, IDH1/2, CEBPA and RUNX1 on overall survival

*Log-likelihood ratio test

Multivariable analyses for EFS and OS including allogeneic HCT in first CR as time-dependent variable revealed no significant impact of ATRA on an ITT basis. However, on a PP basis, ATRA was associated with a significantly (HR, 0.82; *p* = 0.02) better OS (Tables 3, 4, 5, and 6).

**Table 3:** Andersen-Gill regression model with the endpoint EFS analysed on an intention-to-treat basis

	HR	95 % CI	*p* value
Genetic risk according to ELN			
Favorable risk’	0.38	0.31–0.47	<0.0001
Intermediate-2’	1.05	0.86–1.30	0.62
Adverse-risk’	1.80	1.48–2.18	<0.0001
s/t-AML	1.28	1.04–1.57	0.020
Gender (male)	1.35	1.17–1.56	<0.0001
WBC (Median-dichotomized)*	1.29	1.11–1.49	0.001
Valproic acid	1.22	1.02–1.47	0.032
Allogeneic HCT in 1^st^ CR	0.47	0.38–0.60	<0.0001
ATRA	0.99	0.86–1.14	0.87

Variables excluded after limited backward selection in the order of their exclusion: *DNMT3A* mutational status (*p* = 0.82), *ASXL1* mutational status (*p* = 0.70), *RUNX1* mutational status (*p* = 0.56), *FLT3-TKD* (*p* = 0.48), *IDH2* mutational status (*p* = 0.39), *IDH1* mutational status (*p* = 0.32) and age (*p* = 0.10)

*The median WBC of the whole cohort was 12.7 G/L

**Table 4:** Andersen-Gill regression model with the endpoint EFS analysed on a per-protocol basis

	HR	95 % CI	*p* value
Genetic risk according to ELN			
Favorable risk	0.38	0.31–0.47	<0.0001
Intermediate-2’	1.05	0.86–1.29	0.63
Adverse risk	1.79	1.48–2.17	<0.0001
s/t-AML	1.28	1.04–1.58	0.018
Gender (male)	1.35	1.17–1.56	<0.0001
WBC (median-dichotomized)*	1.27	1.10–1.47	0.001
Valproic acid	1.22	1.02–1.47	0.032
Allogeneic HCT in 1^st^ CR	0.47	0.37–0.59	<0.0001
ATRA	0.88	0.76–1.01	0.07

Variables excluded after limited backward selection in the order of their exclusion: *DNMT3A* mutational status (*p*= 0.87), *ASXL1* mutational status (*p* = 0.72), *RUNX1* mutational status (*p* = 0.61), *FLT3-TKD* (*p* = 0.53), *IDH2* mutational status (*p* = 0.35), *IDH1* mutational status (*p* = 0.34) and age (*p* = 0.08)

*The median WBC of the whole cohort was 12.7 G/L

**Table 5:** Andersen-Gill regression model with the endpoint OS analysed on an intention-to-treat basis

	HR	95 % CI	*p* value
Genetic risk according to ELN			
Favorable risk	0.45	0.34–0.58	<0.0001
Intermediate-2	1.03	0.80–1.32	0.84
Adverse risk	1.87	1.50–2.34	<0.0001
s/t-AML	1.32	1.04–1.67	0.021
Gender (male)	1.22	1.03–1.44	0.024
Age (diff. 10 years)	1.23	1.13–1.34	<0.0001
WBC (median-dichotomized)*	1.55	1.30–1.85	<0.0001
Valproic acid	1.36	1.10–1.67	0.004
Allogeneic HCT in 1^st^ CR	0.71	0.58–0.87	0.001
ATRA	0.89	0.76–1.06	0.19

Variables excluded after limited backward selection in the order of their exclusion: *DNMT3A* mutational status (*p* = 0.95), *FLT3-TKD* (*p* = 0.85), *ASXL1* mutational status (*p* = 0.70), *RUNX1* mutational status (*p* = 0.52), *IDH1* mutational status (*p* = 0.29) and *IDH2* mutational status (*p* = 0.13)

*The median WBC of the whole cohort was 12.7 G/L reference group intermediate-1

**Table 6:** Andersen-Gill regression model with the endpoint OS analysed on a per-protocol basis

	HR	95 % CI	*p* value
Genetic risk according to ELN			
Favorable risk	0.45	0.34–0.58	<0.0001
Intermediate-2	1.03	0.80–1.33	0.81
Adverse risk	1.88	1.51–2.34	<0.0001
s/t-AML	1.33	1.05–1.68	0.019
Gender (male)	1.22	1.03–1.44	0.019
Age (diff. 10 years)	1.24	1.14–1.34	<0.0001
WBC (median-dichotomized)*	1.51	1.26–1.81	<0.0001
Valproic acid	1.35	1.10–1.67	0.005
Allogeneic HCT in 1^st^ CR	0.71	0.58–0.88	0.001
ATRA	0.81	0.69–0.96	0.017

Variables excluded after limited backward selection in the order of their exclusion: *DNMT3A* mutational status (*p* = 0.87), *ASXL1* mutational status (*p* = 0.70), *RUNX1* mutational status (*p* = 0.61), FlT3-tKd (*p* = 0.53), *IDH2* mutational status (*p* = 0.35), *IDH1* mutational status (*p* = 0.34) and age (*p* = 0.08)

*The median WBC of the whole cohort was 12.7 G/L reference group intermediate-1

**Fig. 3:**
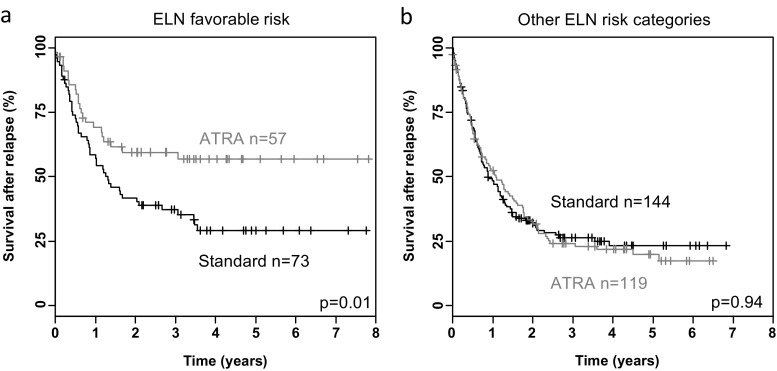
Survival after relapse according to European LeukemiaNet (ELN) classification analyzed on an intention-to-treat basis. **a** ELN favorable-risk group; **b** all other ELN risk groups

Overall, 473 patients relapsed after achieving a first remission either on the protocol (*n* = 397) or after salvage therapy (*n* = 76). According to ELN-risk groups, 130 patients had a favorable risk (CBF-AML, biallelic mutated *CEBPA*, mutant *NPM1*/*FLT3*-ITDneg) and 263 patients had no favorable risk. Of the relapsed patients with favorable risk, 95 patients received allogeneic HCT after relapse; of the 6 patients who had been transplanted in the first CR, 4 patients received a second allogeneic HCT and 2 patients received autologous HCT; and 27 patients were treated with chemotherapy only. Relapsed patients within all other ELN risk groups were treated after relapse with allogeneic HCT (*n* = 116) or chemotherapy (*n* = 78); of 78 patients who had been transplanted in first CR, 24 patients received second allogeneic HCT and 54 patients chemotherapy only. The second CR rates, also including CRs achieved after allogeneic HCT, were not significantly different in STANDARD and ATRA with 65 % (45/69) and 73 % (45/61) in the favorable-risk group, and 48 % (67/141) and 54 % (66/122) in other ELN-risk groups, respectively. In contrast, treatment with ATRA during first-line therapy had a major impact on OS after relapse. Patients in the favorable-risk group had a significantly superior OS after relapse if they had received ATRA (ITT, *p* = 0.006; PP, *p* = 0.02; Fig. 3a) during the first-line therapy, whereas this was not the case in the other ELN-risk groups (ITT, *p* = 0.98; PP, *p* = 0.71; Fig. 3b).

## Discussion

We previously reported that ATRA given in combination with intensive chemotherapy improves survival in older patients with AML [19]. The objectives of this trial were to perform a confirmatory study in a younger patient population and to endorse mutant *NPM1* as a predictive factor for response to ATRA [20].

Induction therapy consisted of idarubicin, etoposide and cytarabine (ICE) with or without ATRA. Based on the early preclinical data, we decided to start ATRA at day 6, that is, after most of the cytotoxic drugs were administered; furthermore, we reduced the daily dose to 15 mg/m^2^ at day 9 to avoid undue toxicity. Due to the open-label character of the study, we implemented in the protocol predefined ITT as well as PP analyses. Our results show that the addition of ATRA to intensive induction therapy is feasible with three days of 45 mg/m^2^ followed by a dose reduction to 15 mg/m^2^ and not associated with relevant additional toxicity. This is in contrast to the results reported in the NCRI AML16 trial, in which continuous high doses of ATRA (45 mg/m^2^) have led to excessive toxicity in 616 randomized patients with a significant increase in the 30-day mortality rate of 20 % in the ATRA arm as compared to 12 % in the standard arm (*p* = 0.005) [38].

Overall, we were not able to show a significant beneficial effect of ATRA on an IIT basis on the primary endpoint EFS and the secondary endpoints CR rate, RFS and OS. In addition, we were also not able to confirm, on an ITT basis, the predictive value of NPM1 mutational status on the beneficial effect of ATRA on clinical endpoints. Thus, our data confirm the results from MRC showing no impact of ATRA on clinical endpoints and in distinct molecular subgroups including mutated *NPM1* with or without *FLT3*-ITD [21]. However, PP analyses revealed some efficacy of ATRA in the total cohort for OS (*p* = 0.03) and for EFS in *NPM1-*mutated AML (*p* = 0.05). Although PP analyses may be biased, these results are supported by multivariable models accounting for important base-line variables and allogeneic HCT which was included as a time-dependent covariable. Thus, to some extent, our previous data on the beneficial clinical effect of ATRA overall [19] as well as in a genetically defined subgroup [20] were supported by the results of the current study.

Our clinical results are supported by recent *in vitro* data in cell lines and primary AML blasts showing the ability of ATRA to induce a significant amount of apoptosis in some (3 out of 11) primary leukemia samples from patients with *NPM1* mutation which was potentiated by combination with ATO [13]. In addition, ATRA alone was also able to induce a marked selective downregulation of NPM1 mutant oncoprotein indicated by the appearance of active caspase-8 fragment and cleaved poly(ADP-ribose)polymerase (PARP). Again, the combination of ATRA with ATO was even more effective [13]. These findings were similarly reported by others showing that ATRA and/or ATO were able to induce proteasomal degradation of mutant NPM1 in AML cell lines or primary samples leading to differentiation and apoptosis [12]. Based on the *in vitro* data, 5 patients with *NPM1*-mutated AML were treated with ATRA/ATO resulting in a transient antileukemic effect [12]. Of note, in contrast to previous *in vitro* data, Martelli et al. showed an increased sensitivity upon treatment with ATRA/ATO 24 to 48 h before treatment with daunorubicin [13]. These data support further exploration of ATRA in combination with ATO and an anthracycline in AML with mutated *NPM1*.

Somewhat surprisingly, the beneficial effect of ATRA in AML with mutated *NPM1* on EFS based on PP analysis did not translate into a beneficial effect on OS. Rather both subpopulations, *NPM1*-wildtype and *NPM1*-mutated AML, contributed to the significantly improved OS (*p* = 0.03) in PP analyses (Fig. 3). As there was no significant impact of ATRA on EFS and RFS, this observation prompted us to analyze outcome after relapse. Most patients received allogeneic HCT after relapse, 76 % in the ELN favorable-risk group and 53 % in the other ELN risk groups. There was a major beneficial effect of ATRA analyzed on an ITT and a PP basis in the ELN favorable-risk group with a significantly better OS after relapse in those patients randomized to and treated with ATRA, whereas no effect was seen in the other ELN risk groups (Fig. 3, Fig. 4). As no significant difference in the second CR rates were evident and most patients received an allogeneic HCT after relapse, the effect of ATRA on outcome after relapse may be explained by preventing further relapses. One hypothesis could be that ATRA modulates antigen presentation in the context of mucosal immunity [39] in patients undergoing allogeneic HCT.

In conclusion, ATRA in combination with intensive induction and consolidation therapy as used in our study can be safely administered. In ITT analysis, no impact on outcome was demonstrated except for a beneficial effect of ATRA in ELN favorable-risk patients. In contrast, in PP analysis, ATRA was associated with an improved EFS in *NPM1*-mutated AML as well as OS in all patients. In addition, ATRA given during first CR impacted on survival in patients with ELN favorable-risk receiving allogeneic HCT after relapse.

## Electronic supplementary material

Below is the link to the electronic supplementary material.

ESM 1 Supplementary Figure 1. Stratified analyses of ATRA on an intention-to-treat basis by genetic risk group according to ELN recommendations and mutational status of *NPM1*, *FLT3*-ITD, *DNMT3A*, *IDH1/2*, *CEBPA*, *RUNX1* on event free survival. *log-likelihood ratio test (PDF 1312 kb)

ESM 2 Supplementary Figure 2. Stratified analyses of ATRA on a per-protocol basis by genetic risk group according to ELN recommendations and mutational status of *NPM1*, *FLT3*-ITD, *DNMT3A*, *IDH1/2*, *CEBPA*, *RUNX1* on event free survival. *log-likelihood ratio test (PDF 1316 kb)

ESM 3 Supplementary Figure 3: Stratified analyses of ATRA on an per-protocol basis by genetic risk group according to ELN recommendations and mutational status of *NPM1*, *FLT3*-ITD, *DNMT3A*, *IDH1/2*, *CEBPA*, *RUNX1* on overall survival. *log-likelihood ratio test (PDF 1316 kb)

ESM 4 (PDF 70 kb)

ESM 5 (PDF 8 kb)
