# Supplementary material for: All-trans retinoic acid as adjunct to intensive treatment in younger adult patients with acute myeloid leukemia: results of the randomized AMLSG 07-04 study
Source: Ann Hematol. 2016 Oct 3;95(12):1931–42. doi: 10.1007/s00277-016-2810-z (PMC5093206; doi:10.1007/s00277-016-2810-z)

Supplementary Figure 1: Event-free Survival, Intention-to-treat analysis

	Events/Patients ATRA	Events/Patients no ATRA	HR (95% CI)	P-value	P-value for interaction*
**ELN risk-group**					**0.422**
favorable	69/139	77/152	0.97 (0.70, 1.35)	0.88	
inter-1	115/151	126/153	0.83 (0.65, 1.07)	0.16	
inter-2	75/90	70/90	1.18 (0.85, 1.63)	0.33	
high	101/117	96/107	1.07 (0.81, 1.41)	0.65	
NPM1					**0.160**
wild-type	274/358	276/362	1.06 (0.90, 1.26)	0.48	
mutated	86/138	102/149	0.82 (0.61, 1.09)	0.17	
***FLT3*-ITD**					**0.997**
negative	287/406	306/423	0.99 (0.85, 1.17)	0.93	
positive	82/102	86/107	0.99 (0.73, 1.35)	0.97	
**Mutated *NPM1*/*FLT3*-ITD negativ**					**0.352**
negative	315/413	321/418	1.03 (0.88, 1.21)	0.69	
positive	47/85	57/94	0.84 (0.57, 1.23)	0.37	
DNMT3A					**0.829**
wild-type	274/378	296/399	1.00 (0.85, 1.18)	0.99	
mutated	89/119	80/109	1.04 (0.77, 1.40)	0.82	
IDH1					**0.796**
wild-type	278/395	300/403	0.94 (0.80, 1.11)	0.47	
mutated	23/29	21/26	1.04 (0.57, 1.88)	0.90	
IDH2					**0.532**
wild-type	270/382	285/384	0.96 (0.82, 1.14)	0.65	
mutated-R140	20/29	24/30	0.72 (0.40, 1.30)	0.27	
mutated-R172	9/11	11/12	0.72 (0.30, 1.76)	0.46	
CEBPA					**0.634**
wild-type	329/442	340/457	1.01 (0.87, 1.17)	0.90	
monoallelic	10/15	10/12	0.88 (0.36, 2.11)	0.77	
biallelic	11/23	17/26	0.72 (0.34, 1.54)	0.40	
RUNX1					
wild-type	261/372	276/378	0.97 (0.82, 1.14)	0.56	**0.612**
mutated	25/32	35/39	0.86 (0.51, 1.44)	0.69	
**All**	**396/544**	**410/556**	**0.99 (0.87, 1.14)**	**0.93**	

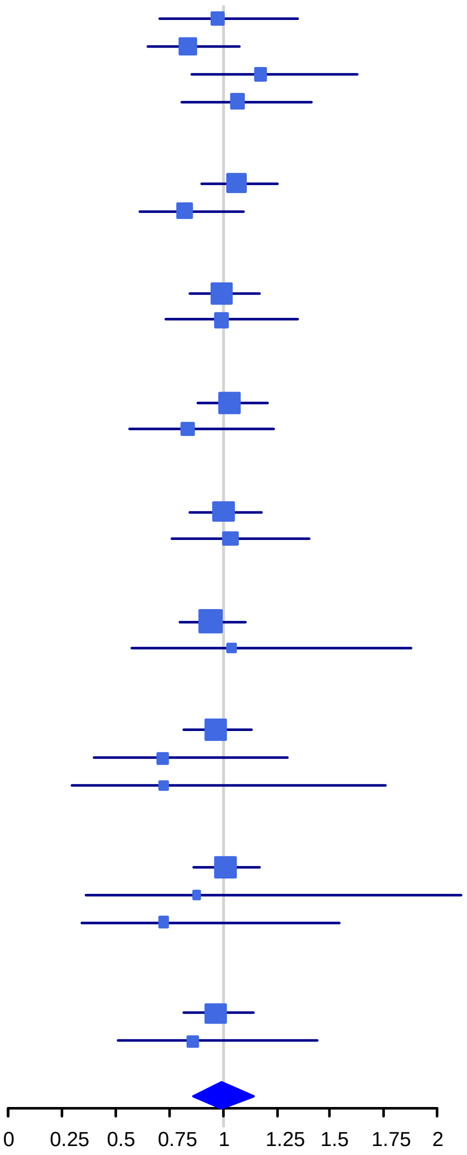

Supplementary Figure 2: Event-free Survival, per-protocol analysis

	Events/Patients ATRA	Events/Patients no ATRA	HR (95% CI)	P-value	P-value for interaction*
**ELN risk-group**					0.579
favorable	71/143	75/148	0.93 (0.67, 1.29)	0.66	
inter-1	113/151	128/153	0.75 (0.58, 0.96)	0.02	
inter-2	72/89	73/91	0.99 (0.71, 1.37)	0.95	
high	98/115	99/109	0.93 (0.70, 1.23)	0.59	
NPM1					0.261
wild-type	266/354	284/366	0.94 (0.80, 1.11)	0.47	
mutated	88/143	100/144	0.75 (0.56, 1.00)	0.05	
***FLT3*-ITD**					0.706
negative	283/409	310/420	0.87 (0.74, 1.03)	0.10	
positive	79/99	89/110	0.93 (0.69, 1.26)	0.66	
**Mutated *NPM1*/*FLT3*-ITD negativ**					0.363
negative	307/409	329/422	0.93 (0.79, 1.08)	0.35	
positive	49/90	55/89	0.75 (0.51, 1.10)	0.14	
DNMT3A					0.600
wild-type	265/374	305/403	0.87 (0.74, 1.03)	0.11	
mutated	91/123	78/105	0.95 (0.70, 1.29)	0.74	
IDH1					0.608
wild-type	270/393	308/405	0.83 (0.70, 0.97)	0.02	
mutated	22/28	22/27	0.97 (0.54, 1.76)	0.92	
IDH2					0.601
wild-type	259/376	296/390	0.84 (0.71, 0.99)	0.04	
mutated-R140	20/30	24/29	0.63 (0.35, 1.14)	0.13	
mutated-R172	11/13	9/10	0.76 (0.31, 1.86)	0.54	
CEBPA					0.782
wild-type	323/443	346/456	0.89 (0.76, 1.03)	0.13	
monoallelic	9/14	11/13	0.74 (0.30, 1.79)	0.50	
biallelic	12/24	16/25	0.74 (0.35, 1.57)	0.44	
RUNX1					0.984
wild-type	252/369	285/381	0.84 (0.70, 0.99)	0.04	
mutated	25/32	35/39	0.86 (0.51, 1.44)	0.56	
**All**	**389/544**	**417/556**	**0.89 (0.77, 1.02)**	**0.09**	

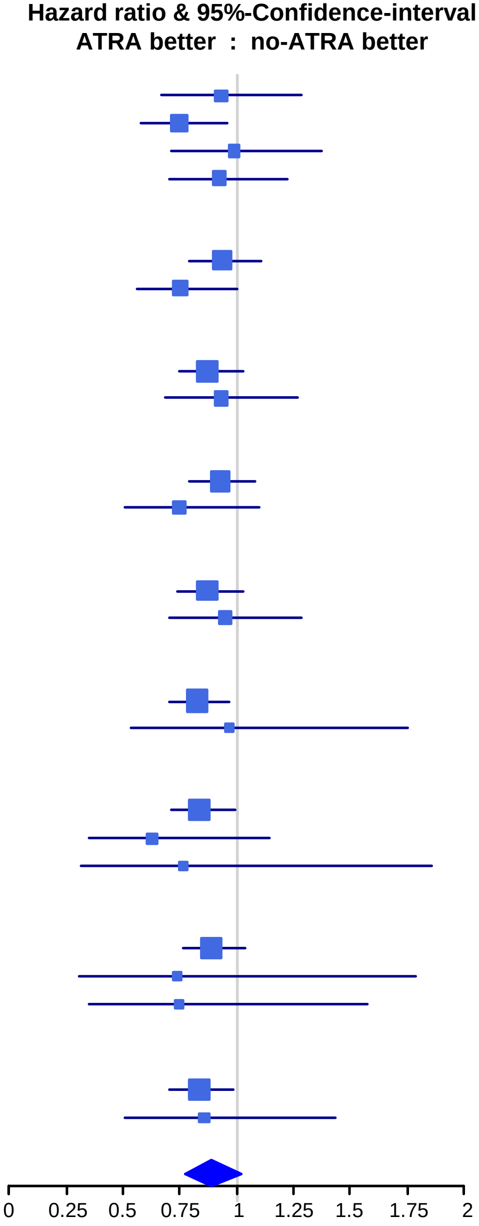

Supplementary Figure 3: Overall Survival, per-protocol analysis

	Events/Patients ATRA	Events/Patients no ATRA	HR (95% CI)	P-value	P-value for interaction*
**ELN risk-group**					**0.866**
favorable	34/143	51/148	0.65 (0.42, 1.01)	0.05	
inter-1	75/151	86/153	0.76 (0.56, 1.04)	0.09	
inter-2	47/89	52/91	0.79 (0.53, 1.17)	0.24	
high	78/115	80/109	0.84 (0.62, 1.15)	0.28	
NPM1					**0.933**
wild-type	174/354	201/366	0.82 (0.67, 1.01)	0.06	
mutated	66/143	73/144	0.83 (0.60, 1.16)	0.29	
***FLT3*-ITD**					**0.493**
negative	185/409	223/420	0.78 (0.64, 0.94)	0.01	
positive	59/99	66/110	0.89 (0.63, 1.27)	0.53	
**Mutated *NPM1*/*FLT3*-ITD negativ**					**0.396**
negative	210/409	234/422	0.86 (0.71, 1.03)	0.11	
positive	31/90	40/89	0.68 (0.43, 1.10)	0.11	
DNMT3A					**0.0796**
wild-type	173/374	222/403	0.75 (0.61, 0.91)	0.0004	
mutated	65/123	51/105	1.09 (0.75, 1.57)	0.65	
IDH1					**0.672**
wild-type	179/393	216/405	0.77 (0.63, 0.93)	0.008	
mutated	15/28	16/27	0.90 (0.44, 1.82)	0.77	
IDH2					**0.914**
wild-type	172/376	211/390	0.76 (0.62, 0.93)	0.008	
mutated-R140	16/30	17/29	0.89 (0.45, 1.76)	0.74	
mutated-R172	5/13	4/10	0.79 (0.21, 2.95)	0.73	
CEBPA					**0.108**
wild-type	219/443	249/456	0.81 (0.68, 0.97)	0.02	
monoallelic	5/14	7/13	0.60 (0.19, 1.89)	0.40	
biallelic	2/24	10/25	0.19 (0.04, 0.89)	0.03	
RUNX1					**0.669**
wild-type	167/369	201/381	0.79 (0.64, 0.97)	0.02	
mutated	19/32	24/39	0.91 (0.50, 1.66)	0.76	
**All**	**264/544**	**298/556**	**0.83 (0.70, 0.98)**	**0.03**	

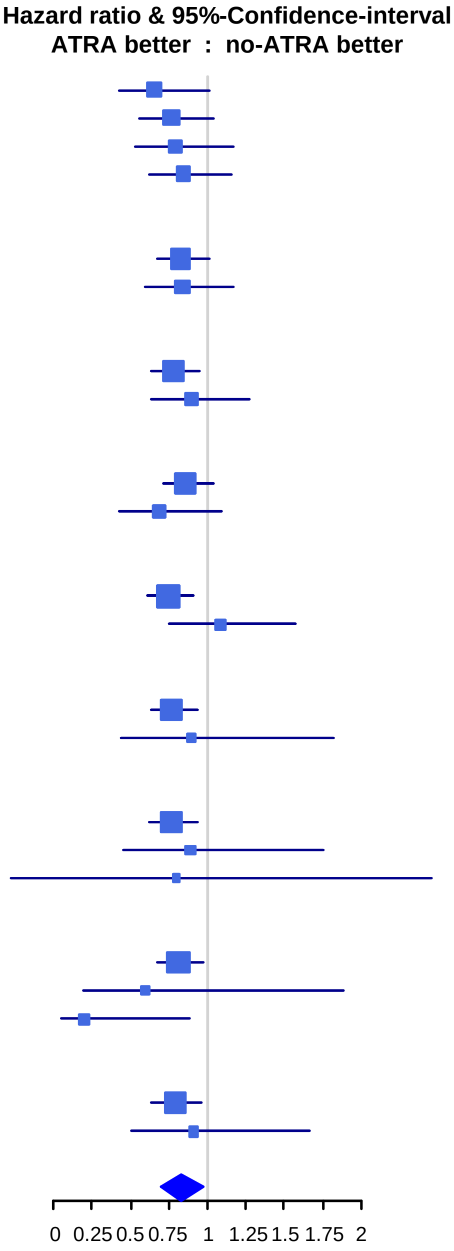

Supplement: Supplementary file 1 — Supplementary Figure 1. Stratified analyses of ATRA on an intention-to-treat basis by genetic risk group according to ELN recommendations and mutational status of NPM1, FLT3-ITD, DNMT3A, IDH1/2, CEBPA, RUNX1 on event free survival. *log-likelihood ratio test (PDF 1312 kb) [file 277_2016_2810_MOESM1_ESM.pdf]
