# Supplementary figures and images for: All-trans retinoic acid as adjunct to intensive treatment in younger adult patients with acute myeloid leukemia: results of the randomized AMLSG 07-04 study
Source: Ann Hematol. 2016 Oct 3;95(12):1931–42. doi: 10.1007/s00277-016-2810-z (PMC5093206; doi:10.1007/s00277-016-2810-z)

Supplementary Figure 2: Event-free Survival, per-protocol analysis

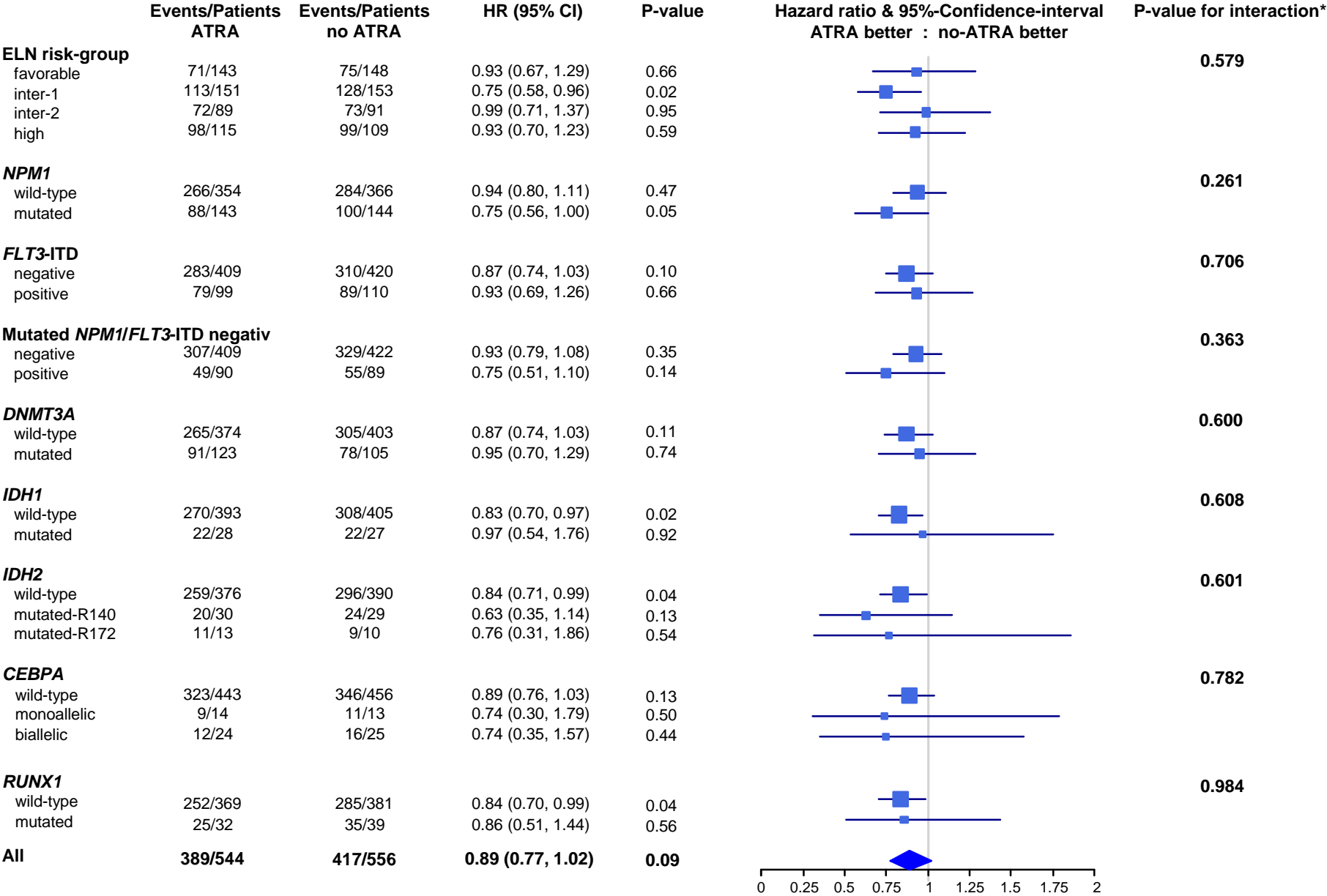

Supplement: Supplementary file 2 — Supplementary Figure 2. Stratified analyses of ATRA on a per-protocol basis by genetic risk group according to ELN recommendations and mutational status of NPM1, FLT3-ITD, DNMT3A, IDH1/2, CEBPA, RUNX1 on event free survival. *log-likelihood ratio test (PDF 1316 kb) [file 277_2016_2810_MOESM2_ESM.pdf]

Supplementary Figure 3: Overall Survival, per-protocol analysis

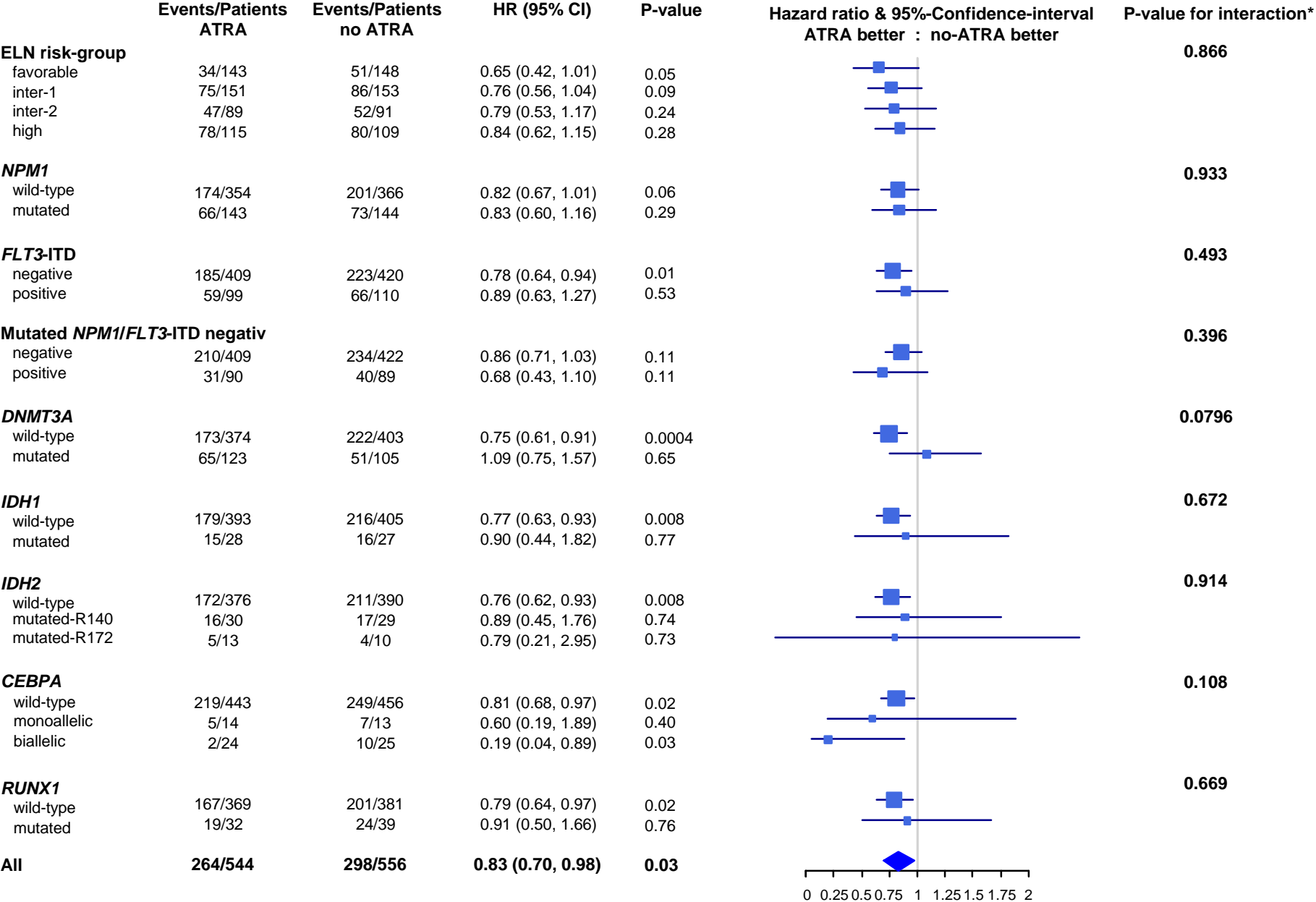

Supplement: Supplementary file 3 — Supplementary Figure 3: Stratified analyses of ATRA on an per-protocol basis by genetic risk group according to ELN recommendations and mutational status of NPM1, FLT3-ITD, DNMT3A, IDH1/2, CEBPA, RUNX1 on overall survival. *log-likelihood ratio test (PDF 1316 kb) [file 277_2016_2810_MOESM3_ESM.pdf]
