# Supplementary material for: All-trans retinoic acid as adjunct to intensive treatment in younger adult patients with acute myeloid leukemia: results of the randomized AMLSG 07-04 study
Source: Ann Hematol. 2016 Oct 3;95(12):1931–42. doi: 10.1007/s00277-016-2810-z (PMC5093206; doi:10.1007/s00277-016-2810-z)
Supplement: Supplementary file 4 — (PDF 70 kb) [file 277_2016_2810_MOESM4_ESM.pdf]

Supplementary Table 1.a
Logistic regression model with the endpoint CR after double induction therapy analysed on an intention-to-treat basis

	OR	95%-CI	p-value
Genetic risk according to ELN			
Favorable-risk‘	3.61	2.16 – 6.02	<0.0001
Intermediate-2’	0.78	0.51-1.20	0.26
Adverse-risk‘	0.40	0.28 - 0.59	<0.0001
Mutated *DNMT3A*	1.64	1.09 - 2.47	0.018
s/t-AML	0.60	0.40 - 0.89	0.012
Male gender	0.70	0.52 - 0.94	0.018
Age (diff. of 10 yrs)	0.85	0.74 - 0.98	0.021
WBC (Median-dichotomized)*	0.72	0.53 - 0.98	0.035
Valproic acid	0.67	0.46 – 0.98	0.040
ATRA	0.99	0.74 - 1.33	0.96

Variables excluded after limited backward selection in the order of their exclusion: *RUNX1* mutational status (p=0.82), *FLT3*-TKD (p=0.61), *ASXL1* mutational status (p=0.30), *IDH2* mutational status (p=0.26), *IDH1* mutational status (p=0.09), VPA (p=0.06). *the median WBC of the whole cohort was 12.7 G/l.

Supplementary Table 1.b
Logistic regression model with the endpoint CR after double induction therapy analysed on a per-protocol basis

	OR	95%-CI	p-value
Genetic risk according to ELN			
Favorable-risk‘	3.52	2.10 - 5.89	<0.0001
intermediate-2’	0.76	0.50 – 1.16	0.21
Adverse-risk‘	0.41	0.28 - 0.61	<0.0001
Mutated *DNMT3A*	1.50	1.01 - 2.24	0.045
s/t-AML	0.62	0.42 - 0.94	0.023
Male gender	0.72	0.53 - 0.96	0.026
Age (diff. of 10 yrs)	0.87	0.74 - 0.99	0.043
Valproic acid	0.68	0.46 – 0.99	0.045
ATRA	1.31	0.98 - 1.76	0.07

Variables excluded after limited backward selection in the order of their exclusion: *RUNX1* mutational status (p=0.81), *FLT3*-TKD (p=0.63), *IDH2* mutational status (p=0.32), *ASXL1* mutational status (p=0.29), *IDH1* mutational status (p=0.11), WBC (p=0.06). *the median WBC of the whole cohort was 12.7 G/l.
‘reference group intermediate-1
