# Supplementary material for: All-trans retinoic acid as adjunct to intensive treatment in younger adult patients with acute myeloid leukemia: results of the randomized AMLSG 07-04 study
Source: Ann Hematol. 2016 Oct 3;95(12):1931–42. doi: 10.1007/s00277-016-2810-z (PMC5093206; doi:10.1007/s00277-016-2810-z)
Supplement: Supplementary file 5 — (PDF 8 kb) [file 277_2016_2810_MOESM5_ESM.pdf]

Supplementary Table 2:
Adverse events grade 3/4 occurring during first induction therapy according to treatment with or without ATRA

	STANDARD<br>n=553<br>No. (%)	ATRA<br>n=538<br>No. (%)	p-value
Allergic reaction	4 (1)	12 (2)	0.04
Cardiac	22 (4)	9 (1.5)	0.03
Coagulation	8 (1.5)	8 (1.5)	0.99
Constitutional symptoms	4 (1)	7 (1.5)	0.38
Dermatology/skin	14 (2.5)	20 (4)	0.30
Gastrointestinal	63 (11.5)	81 (15)	0.09
Hemorrhage/bleeding	14 (2.5)	15 (3)	0.85
Hepatobiliary/pancreas	5 (1)	7 (1.5)	0.57
Metabolic/laboratory	66 (12%)	67 (12.5)	0.85
Neurology	11 (2)	13 (2.5)	0.68
Pain	6 (1)	6 (1)	0.99
Pulmonary/upper respiratory	19 (3.5)	24 (4.5)	0.44
Renal/genitourinary	8 (1.5)	4 (1)	0.38
Vascular	8 (1.5)	7 (1.5)	0.99
Infections	329 (59.5)	341 (63.5)	0.19
Other	13 (2.5)	10 (2)	0.67
